# How do women at increased, but unexplained, familial risk of breast cancer perceive and manage their risk? A qualitative interview study

**DOI:** 10.1186/1897-4287-9-7

**Published:** 2011-09-06

**Authors:** Louise A Keogh, Belinda J McClaren, Carmel Apicella, John L Hopper

**Affiliations:** 1 Centre for Women's Health, Gender and Society, Melbourne School of Population Health, The University of Melbourne, Melbourne, Australia; 2 Genetics Education and Health ResearchGroup, Murdoch Childrens Research Institute, Melbourne, Australia; 3 Centre for Molecular, Environmental, Genetic & Analytic Epidemiology, Melbourne School of Population Health, The University of Melbourne, Melbourne, Australia

**Keywords:** Breast cancer, risk perception, screening, qualitative research, cancer, familial risk

## Abstract

**Background:**

The perception of breast cancer risk held by women who have not had breast cancer, and who are at increased, but unexplained, familial risk of breast cancer is poorly described. This study aims to describe risk perception and how it is related to screening behaviour for these women.

**Methods:**

Participants were recruited from a population-based sample (the Australian Breast Cancer Family Study - ABCFS). The ABCFS includes women diagnosed with breast cancer and their relatives. For this study, women without breast cancer with at least one first- or second-degree relative diagnosed with breast cancer before age 50 were eligible unless a *BRCA1 *or *BRCA2 *mutation had been identified in their family. Data collection consisted of an audio recorded, semi-structured interview on the topic of breast cancer risk and screening decision-making. Data was analysed thematically.

**Results:**

A total of 24 interviews were conducted, and saturation of the main themes was achieved. Women were classified into one of five groups: don't worry about cancer risk, but do screening; concerned about cancer risk, so do something; concerned about cancer risk, so why don't I do anything?; cancer inevitable; cancer unlikely.

**Conclusions:**

The language and framework women use to describe their risk of breast cancer must be the starting point in attempts to enhance women's understanding of risk and their prevention behaviour.

## Introduction

It has been argued that health and risk have replaced illness and disease as the domains of interest for medicine [1,2]. 'The focus is no longer on illness, disability, and disease as matters of fate, but on health as a matter of ongoing moral self-transformation' [1, p172]. Women who have not had breast cancer, but who have a high familial risk of the disease, are a pertinent example of this phenomenon. While they may be physically well, the risk of breast cancer is likely to be a focus of their health care and practice, and there are proven interventions that decrease breast cancer incidence for women at high risk [3-5]. Genetic testing for a disease-predisposing mutation can provide a risk estimate and appropriate risk reduction options can be recommended for women from families in which a mutation is found, but for women from families in which no mutation has been identified this clarity and advice is not available. While there is research on the impact of genetic testing for breast cancer, there is very little research with the group in whom there are inconclusive findings [6] and we know little about how this group manage their risk. There is a clear need to undertake research with women who have a family history that indicates a high risk of breast cancer, but who have not attended a clinical genetics service for counselling.

It is increasingly recognised that risk perception is complex, multi-dimensional, and incompletely understood, and that research has failed to reach consensus on a model for measuring risk perception [7-9]. Collins and Street [10, p1506] argue that risk perception is influenced by 'scientific, psychological, social, economic, and cultural factors' and that while clinicians are more likely to use 'analytic' reasoning, patients are more likely to use 'experiential' reasoning in risk judgements. People actively evaluate health information using the strategies available to them, and risk estimates are subject to this active and ongoing evaluation [11].

Even when people are able to recall an 'objective' risk estimate given to them by a health professional, in their day-to-day deliberations they might not utilise this estimate but instead rely on their own personal 'subjective' risk estimate [12]. Lee [9, p106] summarises; 'risk information is rarely taken up as value-neutral objective truth, but rather risk information is deeply subjective, interiorised against a pre-existing sense of self'.

Despite this, much research on perception of risk for breast cancer utilises uni-dimensional, one or two item measures of risk perception [10]. This type of research has measured the accuracy of perceived risk, compared to an 'objective' risk estimate, and has found that many women either under- or over-estimate their risk [13-19]. Much of the research on risk perception has focused on women who attend a clinical genetics service [2,15,16,20-22] and/or women who know they carry a *BRCA1 *or *BRCA2 *mutation [23]. Two meta analyses of the effect of genetic counselling on risk perception have reported conflicting findings; one reported an increase in accuracy of risk perception due to counselling [24], while the other reported no effect [25]. A review of the literature on risk perception of clinical genetics attendees reported that the evidence for an effect of risk perception on use of health services or the uptake of health related behaviours is 'weak and in some areas contradictory' [20, p55-56].

There are a number of factors known to influence the perceived risk of getting cancer. Tilburt and colleagues [26] reviewed the published research on the association between risk factors and objective measures of risk perception. They identified demographic, clinical, and psychosocial factors associated with risk perception, however, for many of the factors measured, the associations produced variable results. They found the best characterised factors influencing cancer risk perception in high risk patients were distress, worry and depression. It is not clear whether these factors cause higher risk perception or are caused by heightened risk perception. The authors call for improvements in the measurement of risk perception.

It has been argued that the reason for variable and sometimes conflicting findings in this literature is the poor conceptualisation of risk [17,20]. It has been proposed that using objective measures of risk perception is inadequate for understanding this multi-dimensional concept [26]. In order to move beyond treating risk as an objective measure towards treating it as subjective, Zinn [27] calls for a biographical approach (qualitative interviewing in which participants tell their own story and analysis that identifies types) to be applied to research on risk.

In addition, clinic-based samples are likely to be biased towards women who are concerned about their risk, motivated to act, more resourceful and possibly more knowledgeable than the general at-risk population. In order to study risk perception of women at risk of breast cancer in general, these studies are likely to present only a potentially distorted part of the picture. Some research has been conducted on perception of breast cancer using women selected from the general population [11,14,28-31]. Silverman et al. [28] found that women saw breast cancer as more aggressive and more preventable than experts did, and that women tended to be less concerned than experts about the potential harms of screening. Research on the general population, however, is likely to capture a large proportion of women for whom the risk of breast cancer is low. Given that we are not well-equipped to understand how risk perceptions are generated and used by women at high unexplained familial risk of breast cancer, in this study we adopted a qualitative approach to determine how a population based sample of this understudied group of women perceived their risk of breast cancer and how this influenced their screening behaviour.

## Methods

### Setting for study: the Australian Breast Cancer Family Study

The Australian Breast Cancer Family Study (ABCFS) is a population-based case-control-family study of breast cancer. To be eligible for the ABCFS, women had to be diagnosed with a first primary invasive breast cancer when under the age of 60 years between January 1, 1992, and December 31, 1998, and to live in the Melbourne or Sydney metropolitan areas (case subjects). Each case subject was asked to approach their living adult relatives (siblings, parents, and both maternal and paternal grandparents and aunts) to invite them to participate in the ABCFS. All case subjects and selected relatives completed epidemiological questionnaires and provided a blood sample for genetic research. Between 2003 and 2005 a follow-up study of 248 cases diagnosed with breast cancer before the age of 40 years, and 1200 of their relatives, was conducted. Eligibility to take part in this nested qualitative study was assessed prior to contact, and participants who were eligible were asked for their permission to be contacted by the authors (LAK or BJM).

Ethics approval for this study was obtained from the University of Melbourne, Human Research Ethics Committee.

### Subjects

Eligibility was assessed using the following criteria:

Inclusion criteria

• At least one first- or second-degree relative diagnosed with breast cancer before age 50 years

Exclusion criteria

• Over the age of 70 years

• Prior diagnosis of breast or ovarian cancer

• Known to be a *BRCA1 *or *BRCA2 *mutation carrier

• Not living within the greater Melbourne area

### Recruitment

ABCFS participants who agreed to be contacted for this study were mailed a Participant Information Sheet and Consent Form, and were contacted by either LAK or BJM to arrange an interview time and place. Informed consent was obtained in person, prior to the commencement of the interview. Recruitment continued until saturation of the main themes was reached.

### Data collection & analysis

The data collection consisted of an audio recorded, semi-structured interview on the topic of breast cancer risk and screening decision-making in the area of breast cancer prevention. Women were encouraged to tell the story of the breast cancer in their family in their own words, to describe their own logic about breast cancer risk, and were probed about their perception of risk, using comments such as 'really?', 'tell me more about that,' and 'explain that to me.' Sometimes risk perception had to be raised in a number of different ways in order to get a clear sense of a woman's perception. The interviews were transcribed verbatim, identifying details removed and participants were assigned pseudonyms. Interviews were conducted in homes, workplaces or cafés and lasted between 45 and 90 minutes. The signed Consent Form was collected on the day of the interview. The interview started with questions about the participant's family, background, their experience of cancer in the family, and then went on to explore: perceived causes of cancer, personal risk of cancer, risk-reducing behaviour, risk-increasing behaviour, prevention of cancer, use of health services, use of mammography and breast self-examination.

While these women would have completed epidemiological questionnaires as part of the ABCFS, the questionnaires were not used as a source of data for this analysis. In order to understand women's decision making processes around risk and screening, we chose to rely on women's own version of their behaviour and decisions as described during the interview.

Thematic analysis was performed by reading and re-reading transcripts of the interviews to identify common themes. Together BJM and LAK developed the main themes, and data was double-coded to ensure reliability of coding. Data in the theme 'risk perception' was further analysed to categorise women by way they perceived and managed risk. Other themes will be reported elsewhere. NVivo qualitative data analysis software was used to manage the data analysis process [32].

## Results and discussion

Of the 27 women who agreed to be contacted for this study, 24 completed an interview. Their demographic characteristics are summarized in Table 1. Unlike the majority of studies on risk perceptions for breast cancer, this was not a highly-educated sample, with only 17 per cent having graduated from a university. The majority of participants were over 45 years old and had at least one first-degree relative diagnosed with breast cancer before the age of 50 years.

**Table 1:** Demographic characteristics of interview participants

Characteristics		Number (%) n = 24	
Age	35-45	6	(25)
	46-55	15	(62)
	56-70	3	(13)

Family History	One FDR* > 50	5	(21)
	One FDR < 50	15	(62)
	More than one FDR < 50	4	(17)

Country of birth	Australia	20	(83)
	Other	4	(17)

Education	University graduate	4	(17)
	Other training	13	(54)
	Neither	7	(29)

*First-degree relative

During analysis it became clear that women's personal or perceived risk was not a concept that could easily be separated from their emotional response to their perceived risk or their practical response to their perceived risk. Therefore these three interlinked concepts were analysed together. This analysis revealed what we have called 'risk management styles'. Risk management style takes into account not only what women said about their risk, but also how they felt about their risk, what they said they did about their risk.

Women were classified into one of five risk management styles: don't worry about cancer risk, but do screening; concerned about cancer risk, so do something; concerned about cancer risk, so why don't I do anything?; cancer inevitable; and cancer unlikely. Each group will be explained and illustrated with quotes. In addition, for each woman we report; age, family history, description of their risk, mammogram frequency and health practitioners accessed for breast cancer risk.

In Australia, screening mammograms can be requested by a general practitioner, a specialist or accessed free of charge to women over the age of 40 years through the Government funded *BreastScreen *service (screening is targeted to women 50 years and older, but available to women aged 40-49 years). Women at high-risk are also eligible for Government funded breast ultrasound and MRI, but very few women in this study had accessed either of these breast cancer screening modalities. Women were coded using a number ranging from one to four to indicate the types of health services they used for managing their breast cancer risk (Table 2). The majority of women in this sample did not have a regular specialist to manage their risk, but were relying solely on their General Practitioner (GP), or a GP and occasional specialist as required.

**Table 2:** Summary of the range of health services women accessed to manage breast cancer risk

Code	Health services accessed to manage breast cancer risk	Number (%) n = 24
1	Regular GP and regular specialist	6 (25)

2	Regular GP and occasionally specialist	5 (21)

3	Regular GP only,	12 (50)

4	No Regular GP	1 (4)

### Group 1: Don't worry about cancer risk, but do screening

There were seven women who described this style of managing their unexplained familial risk. These women could all describe that intellectually they knew that they were at higher than population risk due to their family history, but rather than this being a cause of concern, they claimed not to worry about their breast cancer risk. They also all claimed to routinely undergo screening (including mammography, breast self-examination and clinical breast exams). Some had yearly mammograms, others two-yearly, and all began having mammograms before the age of 50 years. Details of each participant displaying this risk management style are summarized in Table 3. Their description of their risk is also included in Table 3, but considered alone this description does little to inform us about how the participant perceives and manages risk.

**Table 3:** Members of Group 1 'Don't worry about cancer, but do screening'

Pseudonym	Age	Family members with breast cancer, age of Dx	Description of risk	Mammogram		Health Provider^a^
				
				Frequency	Age at first	
Maria	45	Sister, 30	*Same as most other people*	2 yearly	34	3

Eva	47	Sister, 33	*No more risk than any other woman*	2 yearly	36	3

Sally	50	Sister, deceased, 37	*More than 1 in 11*	Yearly	30	1

Chloe	53	Sister, deceased, 41	*A fair bit higher than the population*	2 yearly	35	4

Marcia	55	Sister, 42	*A high risk of getting it*	Yearly	43	2

Katrina	56	Sister, deceased, 41 Mother (bowel cancer) deceased, 70	*In a high risk category*	Yearly	45	3

Maggie	66	Daughter, 24 Sister, deceased, 42 Mother and two aunts, deceased	*Couldn't specify*	Yearly	40	1

a: 1: Regular GP and regular specialist, 2: Regular GP and occasionally specialist, 3: Regular GP only, 4: No Regular GP

Maggie was asked how she imagined her own risk of getting breast cancer,

*I don't put a lot of thought into that actually. It doesn't sort of worry me or anything. I just go and have my checks and when I have them I just hope, you know, until you get the results you're a bit, not toey, but you know, you're glad when you hear the results. ...It sort of doesn't worry me--it's not a thing that's on my mind like that ... I mean, it doesn't keep me awake or I don't stress over it*.

Similarly, Marcia describes her perception of her own risk of getting breast cancer,

*I know that if you've got a close family member who's died of breast cancer, you've got a higher risk of getting it, but, no, I don't think about it ... No. I try and be really conscientious myself ... I do all the checks. You know, so as soon as [sister] was diagnosed I started to have mammograms. ... at least if you feel that you've, been conscientious about, about monitoring all those things then, then that kind of removes that element of, of concern. Um, but other than that I'm not particularly tuned into it I don't think*.

She goes on to say,

*I don't think about it for eleven, eleven months and three weeks, and stupidly, I suppose I, I don't think about it until I go for the mammogram ... And I'm reasonably conscientious - in case you think I am absolutely hopeless - so I do the examination each month and, you know, or, as soon as someone starts talking about it. Um, but I don't worry about it otherwise*.

This group all had one or two first-degree relatives diagnosed with breast cancer before the age of 50 years and, while they could identify that they were at higher than average risk, they were keen to emphasise that they were not overly concerned about their risk. Each was doing regular screening, based on the advice they had been given by a health professional. It was not possible to determine whether it was the freedom from concern that allowed them to pursue regular mammograms, or whether the regular mammograms alleviated anxiety, although Marcia indicates that being conscientious about monitoring her body '*removes that element of concern*,' so for her, it may be that screening does alleviate anxiety. There was evidence that these women sensed that they may be judged on the basis of their response to their risk of cancer; for worrying either '*too much*' or '*too little*'; or for not doing the recommended screening. These women wanted to show that they had achieved the 'right' balance, by not worrying excessively, and by adhering to the appropriate screening practices.

### Group 2: Concerned about cancer risk, so do something

Six women were more comfortable expressing concern about their risk of breast cancer, and their response to the risk and concern was to '*do something about it*'. For all of these women, yearly mammograms (starting before age 50) were performed in order to detect cancer at an early stage, but they also described the other strategies they used to reduce their risk. They talked about having a healthy lifestyle, reducing stress and monitoring their bodies through breast self examination. They could be described as '*vigilant' *in the way they managed their risk, and some of these women used this term to describe themselves. For this group, the vigilance was inspired by their concern about their cancer risk, and the belief that their actions could reduce their risk of breast cancer. They admitted their concern about their risk, unlike the women in Group 1 who were keen to point out that they did not worry about cancer. Details of each participant displaying this risk management style are summarized in Table 4.

**Table 4:** Members of Group 2 'Concerned about cancer risk, so do something'

Pseudonym	Age	Family members with breast cancer, age of Dx	Description of risk	Mammogram		Health provider^a^
					
				Frequency	Age at first	
Bev	42	Sister, deceased, 38	*Guess 50%*	Yearly	30	1

Sandra	47	Mother, 72 Cousin, deceased, 43	*Up there*	Yearly	36	3

Jessica	48	Sister, deceased, 40 Grandmother, aunt, 60+	*Not much higher then anyone else*	Yearly	37	1

Kerrie	49	Sister, deceased, 35 Mother, 60+	*Definitely higher risk*	Yearly	38	3

Jane	49	Sister, deceased, 38 Mother, aunt 60+	*A chink higher than population risk*	Yearly	37	1

Vera	70	Mother, 68 Cousin, deceased, 35	*Middle road*	Yearly	49	1

a: 1: Regular GP and regular specialist, 2: Regular GP and occasionally specialist, 3: Regular GP only, 4: No Regular GP

Bev was asked to describe her risk of cancer,

*I always -- you know, I check my breasts ... I'm not um, you know, once a month and all that sort of stuff, but I do check them and I have regular -- the last mammogram I had they did an ultrasound. I hadn't had an ultrasound before that, and everything came back clear and um, so I'm pretty vigilant with it*.

Instead of describing her risk, Bev goes straight into a description of the things she does to manage risk. Vera had a similar response when asked to describe her own risk of cancer,

Well I try and do the right thing ... as far as don't drink too much ... try and look after my diet, so I feel that perhaps it's sort of middle road, I wouldn't class myself as a high risk I don't think

LAK: So what do you think puts people at that higher risk?

*Well perhaps if they don't go and have their mammograms. Um, ah, don't look after their health in general*.

These two issues of risk and risk management were very closely linked for the women in this group to the extent that, for both Vera and Bev, the two concepts were interchangeable. For them, appropriate prevention behaviour had the effect of reducing their perceived risk. Therefore the concern they felt about their breast cancer risk was alleviated by '*vigilant*' prevention behaviour like mammography and breast self examination. All women in this group conducted yearly mammography to manage the risk, even though not all had a first-degree relative diagnosed before the age of 50 years, and five of the six women were in their forties. Four of the woman in this group were also coded as 'one' for health services use, as they all had both a regular GP and a regular specialist, while two had only a GP, and accessed mammograms through their GP or *BreastScreen*.

### Group 3: Concerned about cancer risk, so why don't I do anything?

Four women expressed concern about their risk of breast cancer due to a family history; but almost in the same breath asked themselves, '*why don't I do anything?*' They said they knew they should be having mammograms, and gave a range of reasons for not doing so. Details of each participant displaying this risk management style are summarized in Table 5. Each woman had a regular GP, and had at least been to a specialist once in the past regarding her breast cancer risk, and had been advised to have regular mammograms, but none was currently seeing a specialist on a regular basis. Despite thinking her risk was '*one in three*', Trish felt embarrassed about her weight, and did not want a mammogram until she could lose weight. She had not had a mammogram for seven years. Isabelle described being in shock after her brother's death, and also being too busy to have a mammogram. Two women said that they '*would know*' if they had breast cancer and gave this as a justification for not having mammograms. However, all the women in this group expressed disappointment in themselves for not being more vigilant (referring to themselves as '*slack*' or '*stupid*'). They all had had their first mammogram at a very young age (between the ages of 22 and 34 years) but none had managed to continue with routine mammograms since then, and described themselves as '*not doing enough*.'

**Table 5:** Members of Group 3 'Concerned about cancer risk, so why don't I do anything?'

Pseudonym	Age	Family members affected breast cancer, age at Dx	Description of risk	Mammogram		Health provider^a^
					
				Frequency	Age at first	
Trish	35	Sister deceased, 26 Aunts and uncles, 60+	*One in three*	7 years since last mammogram	22	2

Isabelle	40	Brother (bowel cancer), deceased, 37 Sister, 31 Mother, 68	*High*	A few years since last mammogram	25	2

Anne	41	Sister, deceased, 37 Mother other cancer 2 × sisters other cancer in childhood	*Above average*	Had two mammograms some time ago	27	2

Connie	47	Sister, 36	*Same as population*	A while since last mammogram	34	2

a: 1: Regular GP and regular specialist, 2: Regular GP and occasionally specialist, 3: Regular GP only, 4: No Regular GP

Anne describes her feeling about her chances of getting cancer as,

*I'm not paranoid that I'm going to get it, but I'm still -- I'm not, I'm not sitting back and thinking ... I don't think I'll get it*,

But she described herself as '*slack*' about prevention behaviour. Anne felt that she was '*pretty in tune*' with her body and would '*know when there's something's wrong*'. She describes her approach;

And maybe that's why I'm a bit slack, maybe I think I'll know when there's - and actually I think that's what it is, I'll know when there's something wrong with my body...

Connie seemed to be very concerned about breast cancer, so LAK asked her how often cancer would come to mind,

*I think weekly ... Which is a concern isn't it? That I don't do anything about it. Just worrying about it*.

While Anne was able to articulate a reason for not doing her screening, and Connie could not, both were concerned about their risk of breast cancer, and both were able to recognise and articulate the inconsistency between their thoughts and behaviour. Anne described herself as '*not paranoid*' but also as not thinking '*I don't think I'll get it*.' Here she is articulating the range of risk perceptions available to her, and identifying herself somewhere in the middle of the continuum. Connie admits to thinking about breast cancer weekly, and to worrying about it. Along with their concern about breast cancer was the feeling that they were not doing what was expected of them to manage their risk. The women in this group were able to admit that they had not adhered to the 'ideal' model for managing their breast cancer risk, which would involve more vigilance (as demonstrated in the previous group), and all attempted to explain or justify their behaviour. Once again, their comments reveal women's sense that there are social expectations about the appropriate management of breast cancer risk, and that women would be judged (and would also judge themselves) according to this perceived expectation.

### Group 4: Cancer inevitable

For five participants, their familial history of cancer was interpreted as meaning that they were going to get breast cancer. They did not describe a level of risk, but instead talked about the inevitability of breast cancer. For some this meant they had regular mammograms in order to pick up the cancer early, for others a sense of fatalism led them to neglect screening. Details of the five participants displaying this risk management style are summarized in Table 6. None had attended a specialist about their breast cancer risk.

**Table 6:** Members of Group 4 'Cancer inevitable'

Pseudonym	Age	Family members affected breast cancer	Description of risk	Mammogram		Health provider^a^
					
				Frequency	Age at first	
Sue	41	Sister, deceased, 37 Mother, cervical, 59 Grandmother, 50+ Aunt, 36 Aunt, 34	*If I'm going to die it's going to be of breast cancer*	10 years between mammograms	28	3

Raelene	47	Cousin, 35 Dad, other cancer, deceased, 69	*I think I'll probably die of some kind of cancer*	Will start at age 50 with Breast screen	N/A	3

Alexandra	51	Sister, deceased, 32 Sister deceased, 39 Sister, 35 Grandmother, deceased, 45 Dad, other cancer, deceased, 70	*Time bomb*	Every couple of years	35	3

Bobbie	51	Sister, deceased, 41 Mum, deceased, 73 Dad, deceased, 60+ Grandmother, other cancer 60+ Aunt other cancer 60+	*You will die of something and we just expected that that's what we're going to die of*.	Yearly	38	3

Clare^b^	55	Cousin, 35 Mum, deceased, 59 Father, other cancer, deceased, 70	*I got it into my head that I was going to get it*	Yearly	40	3

a: 1: Regular GP and regular specialist, 2: Regular GP and occasionally specialist, 3: Regular GP only, 4: No Regular GP

b: Felt she had changed her risk perception after speaking to a genetic counsellor and no longer has a sense of fatalism.

Alexandra described her risk of breast cancer as a 'time bomb' and therefore felt that there was little point doing anything to manage her risk. LAK asked her if she thought her smoking would influence her risk of getting breast cancer,

Oh, probably ... But I figure it's probably gonna happen anyway, sooner or later - with our family history. I just go "oh, well, if it's gonna happen, it's gonna happen"

Sue was distressed about her risk of breast cancer, and felt it was inevitable for her and her remaining sisters. This left her unable to undergo regular mammograms due to fear that it would lead to a cancer diagnosis.

*Sue    Well I think that breast cancer's going to get us all in my family. All the girls in my family*.

LAK   Do you think definitely?

*Sue    Um, yeah. I'm very bitter about breast cancer*.

//

LAK   How much do you think about the risk of cancer?

*Sue    It's there all the time. I think about my kids growing up without a mum everyday*.

LAK   Really?

Sue   All the time. Sorry. (crying)

//

LAK   So what about mammograms?

Sue    Oh, I've been slack with them. It's always the thing that I'm scared of the results but I had one a few weeks ago

LAK   How long would it have been since you had one before that?

*Sue   Ten years*.

Women in this group were convinced that they would get cancer. Rather than seeing breast cancer as an event with a level of probability attached to it, they saw breast cancer as a certainty in their life. This certainty had the potential to lead to fatalism and the sense that nothing they did would make any difference (for Sue and Raelene). However, three women in this group (Alexandra, Bobbie and Clare) were able to combine the sense that cancer was inevitable with regular screening mammography, showing the different consequences, depending on how they interpreted and responded to their risk perception.

### Group 5: Cancer unlikely

Finally, two women were convinced that they were not going to get breast cancer, despite their family history. Both women were uneasy about sharing their risk perception during the interview and prefaced their statements with the adjective '*silly*'. Neither had attended a specialist regarding their breast cancer risk. Details of the two participants displaying this risk management style are summarized in Table 7.

**Table 7:** Members of Group 5 'Cancer unlikely'

Pseudonym	Age	Family members affected breast cancer	Description of risk	Mammography		Health provider^a^
					
				Frequency	Age at first	
Carly	51	Sister, 38 Sister, deceased, 47 Mother, deceased, 65	*Do not think I will get cancer*	Recommended yearly, last mammogram 2 years ago	38	3

Lorna	53	Sister, deceased, 46 Aunt, 50+	*Do not think I will get cancer*	2 yearly	35	3

a: 1: Regular GP and regular specialist, 2: Regular GP and occasionally specialist, 3: Regular GP only, 4: No Regular GP

Carly stated at several different points during the interview that she did not think that she was going to get cancer,

I have...no this sounds silly, and I'll fall over if it happens...but I feel the, the three out of four got it, and for some reason I was blessed that I didn't get it, and for a long time there I felt so guilty -- that I didn't get it, you know

//

*No, I don't feel that I'll get it. But when we moved down here and I went to the doctor and he started asking about family history and then sent me off to get a mammogram and speak to a breast specialists and things like that, but that was probably two years ago and I haven't done anything else*.

//

*I check myself every now and then, but not properly because I send positive thoughts*,

//

*So no, I don't think I'll get it*.

Lorna also thought she was not at risk of breast cancer, but she still had mammograms.

*Um, [sister] tended to take after mum's side of the family, I take after dad's side of the family. That's probably a very silly comment, but I mean I do have mammograms, but breast cancer is something that's never worried me*.

Both Lorna and Carly were reluctant to reveal their belief that they were not going to get cancer, as they appeared to understand that this was not a socially acceptable position for women with a strong family history of breast cancer. However, both were able to articulate the reason for their belief, and despite describing a similar perception of risk, they responded to it differently, one having mammograms, the other avoiding them when possible.

### Perceiving and managing risk

Given the high level of unexplained familial risk participants were managing, it is of concern that half of these women had only ever seen a general practitioner about their risk and that only a quarter were consulting a specialist on a regular basis. The evidence of an effect of genetic counselling on risk perception or cancer worry is contradictory [24,25], however, as identified by Bennett et al. [33], 'contact between patients and health professionals and services affords a key opportunity to intervene.' Women in this study had not discussed their risk with a genetic counsellor. Women therefore relied heavily on their own interpretation of their breast cancer risk and often described themselves as 'silly,' 'slack' or 'stupid.' Despite their lack of confidence in their own risk perception, and for some, a concern about staying within the bounds of what they thought was socially acceptable, all were able to describe their perceptions and their decision-making processes in detail, and were able to reflect on their own decision making and behaviour.

While women are generally able to tick a box, or mark a line to indicate their risk perception, the foregoing analysis suggests that they may have revealed only a small part of the whole picture. Risk perception was a layered concept for women in this study; their understandings of heredity, risk factors, genetics and popular discourses about cancer were overlaid with their sense of the social expectations about how they should interpret and manage their breast cancer risk; they felt they must justify thoughts and ideas that were inconsistent with their actions. In addition, even the interpretation of statistics was personal and varied from woman to woman. A woman may consider herself at a 'one in three' chance of getting breast cancer, but she may believe that she is not the 'one' who will get cancer in her family, while another woman may interpret a 'one in three' chance of getting cancer, to mean that she will therefore definitely die of breast cancer.

While the tendency to see risk as a binary phenomenon (either it will or won't happen) has been reported by others [17,34], here we were able to put the group who interpreted their risk in this way in the context of the other risk management styles expressed by women. We have found that the side of the binary state that women identify with (I will or I will not get cancer) does not predict their screening behaviour. However, those women who thought their cancer was inevitable appeared most in need of support to manage their risk. None of these women had a regular specialist, despite exhibiting strong family histories of breast cancer and significant distress in some cases.

While other qualitative research has found that women living at high risk tend to be either making lifestyle or health care adjustments to reduce risk [21,35,36], we have found a greater range of risk management styles in our study, including those who do not know why they 'don't do anything.' The greater range of responses to risk revealed here is likely to be due to the fact that we were able to recruit outside the more motivated clinic-based sample to include women who were managing their familial breast cancer risk without the support of specialists, or genetic counselling or testing. This group are traditionally hard to access for qualitative research, but working within a population-based case-control-family study allowed us access to this under studied group.

## Conclusions

While there is agreement that understanding risk perception is an essential task if we are to assist women to manage risk, and make an informed decision about screening and prevention practices [19], there is also agreement that we currently do not have a consensus on effective ways to measure risk perception [7-9]. Risk is more complicated than many of the instruments developed to measure objective risk perception are able to capture, as people perceive risk in the context of their day-to-day lives, within their families, using the evidence they have been provided with, and the everyday reasoning tools available to them [9].

The analysis presented here is an attempt to answer the question; how do women perceive and manage unexplained familial breast cancer risk? We found no direct positive association between the number of family members diagnosed with breast cancer and perceived risk. We found that women's statements about their perceived risk (e.g. '*middle road*' or '*one in three*') provide only a portion of the information needed to understand their risk perception. Instead, risk perception, the emotional response to the risk perception and the practical response to risk perception are all interconnected and must be considered together.

We recognised five 'risk management styles' expressed by women at high unexplained familial risk of breast cancer, and while this is unlikely to be an exhaustive list, it is a useful starting point. We argue that women's own way of making sense of their experience must continue to be considered legitimate and worthy of study.

Given the main source of advice for many of these women was their general practitioner, this research also highlights the importance of GPs being adequately informed and resourced to manage risk assessment and the anxiety that will be associated for some women, and if not, for them to refer women to a more appropriate service and encourage them to attend. Women in this study who seemed best able to manage their risk were those who had a regular specialist, which may indicate that they were well informed and therefore able to negotiate the health care system effectively to arrange to see a specialist in the first place, or it may have been the interaction with the specialist that was facilitating and endorsing appropriate screening and management of risk.

## Competing interests

There are NO commercial associations that pose or create competing interests with the information presented in this manuscript.

## Authors' contributions

LAK conceived of the study, and participated in its design and coordination, conducted interviews, analysed the data and drafted the manuscript. BJM conducted interviews, contributed to the analysis and interpretation of the data, and commented on drafts of the manuscript, CA contributed to the study design, oversaw recruitment and commented on drafts of the manuscript, JLH contributed to study design, and interpretation of data analysis and commented on drafts of the manuscript. All authors read and approved the final manuscript.
